# Correlational structure of ‘frontal’ tests and intelligence tests indicates two components with asymmetrical neurostructural correlates in old age

**DOI:** 10.1016/j.intell.2014.05.006

**Published:** 2014-09

**Authors:** Simon R. Cox, Sarah E. MacPherson, Karen J. Ferguson, Jack Nissan, Natalie A. Royle, Alasdair M.J. MacLullich, Joanna M. Wardlaw, Ian J. Deary

**Affiliations:** a Brain Research Imaging Centre, Neuroimaging Sciences, University of Edinburgh, UK; b Centre for Cognitive Ageing and Cognitive Epidemiology, University of Edinburgh, UK; c Department of Psychology, University of Edinburgh, UK; d Geriatric Medicine, University of Edinburgh, UK; e Scottish Imaging Network, a Platform for Scientific Excellence (SINAPSE) Collaboration, UK; f Endocrinology Unit, University of Edinburgh, UK

**Keywords:** Ageing, Frontal lobes, Intelligence, Neuropsychology, MRI

## Abstract

Both general fluid intelligence (*g*_f_) and performance on some ‘frontal tests’ of cognition decline with age. Both types of ability are at least partially dependent on the integrity of the frontal lobes, which also deteriorate with age. Overlap between these two methods of assessing complex cognition in older age remains unclear. Such overlap could be investigated using inter-test correlations alone, as in previous studies, but this would be enhanced by ascertaining whether frontal test performance and *g*_f_ share neurobiological variance. To this end, we examined relationships between *g*_f_ and 6 frontal tests (Tower, Self-Ordered Pointing, Simon, Moral Dilemmas, Reversal Learning and Faux Pas tests) in 90 healthy males, aged ~ 73 years. We interpreted their correlational structure using principal component analysis, and in relation to MRI-derived regional frontal lobe volumes (relative to maximal healthy brain size). *g*_f_ correlated significantly and positively (.24 ≤ *r* ≤ .53) with the majority of frontal test scores. Some frontal test scores also exhibited shared variance after controlling for *g*_f_. Principal component analysis of test scores identified units of *g*_f_-common and *g*_f_-independent variance. The former was associated with variance in the left dorsolateral (DL) and anterior cingulate (AC) regions, and the latter with variance in the right DL and AC regions. Thus, we identify two biologically-meaningful components of variance in complex cognitive performance in older age and suggest that age-related changes to DL and AC have the greatest cognitive impact.

## Highlights

• We assess overlap between ‘frontal’ tests and fluid intelligence (*g*_f_) in old age.
• We also examine how units of common score variance relate to frontal lobe volumes.
• *g*_f_-common variance was linked with variance in some left frontal lobe regions.
• *g*_f_-independent variance was linked with homotopic right frontal regions.
• We identify two biologically-meaningful components of complex cognition in old age.

## 1 Introduction

The brain's frontal lobes support a range of complex cognitive functions and comprise several densely interconnected, but structurally heterogeneous sub-regions. They are a major focus of interest in both neuropsychology and differential psychology. Here, we empirically bring together assessments from these two psychological approaches and relate them to regional volumes from the brain's frontal lobes in older age.

Tasks have been developed in the domain of experimental neuropsychology to elicit specific frontal brain activation patterns (from functional imaging) or be sensitive to behavioural profiles caused by focal frontal lesions (Stuss & Levine, 2002), which we shall call ‘frontal’ tests. The emerging picture from neuropsychology, based on lesion studies and functional neuroimaging, indicates modularity for frontal lobe structure–function mapping whereby distinct regions, whilst densely interconnected, each make discrete contributions to performance on tests of complex cognition. This has led to a broad segregation of function between dorsal and ventral frontal regions (MacPherson et al., 2002, Phillips and Della Sala, 1998, Sarazin et al., 1998, Steele and Lawrie, 2004, Stuss et al., 1995).

Differential psychology aims to understand the nature and causes of individual differences in psychological traits and states, including cognitive abilities. Normal healthy individuals who perform well in one cognitive domain (such as processing speed, memory and reasoning) also tend to perform well in another (Carroll, 1993). Current neurobiological models of general intelligence (*g*; a central concept in differential psychology) indicate a central role for the functioning of dorsolateral and cingulate, but not ventral regions of the frontal lobes (Duncan, 2010, Jung and Haier, 2007). More recently however, the contribution of ventral regions to intelligence has also been suggested, using voxel-based morphometry (Colom et al., 2009; Narr et al., 2007) and lesion-based mapping (Barbey et al., 2012, Gläscher et al., 2009). Therefore, the relationships between the cognitive tests used in neuropsychology and differential psychology are of interest.

The frontal region of the brain is particularly susceptible to the effects of age. Its gross volume, cortex (volume and thickness) and white matter (volume and diffusion-based measures of integrity) show disproportionate age-related decreases compared to other parts of the brain (Burzynska et al., 2012, Driscoll et al., 2009, Fjell et al., 2009, Sullivan and Pfefferbaum, 2007). Increasing age is also accompanied by a decline in complex cognitive functioning indexed by some frontal tests (Kemp et al., 2012, Lamar and Resnick, 2004, MacPherson et al., 2002) and also general fluid intelligence (*g*_f_; Deary et al., 2009, Salthouse, 2004). Despite the interest that differential psychologists and neuropsychologists share in the frontal lobes of the brain and how they age, there are few comparisons of scores from the tests produced by these two areas of psychology (Davis, Pierson, & Finch, 2011). It is important to capture all aspects of cognitive ageing if we are to understand its nature and determinants, but two key issues of validity levelled at frontal tests (Rabbitt, Lowe, & Shilling, 2001) have significantly hampered research on this issue in the cognitive ageing literature: vagueness of conceptual boundaries and uniqueness of theoretical construct.

### 1.1 Vagueness of conceptual boundaries

The cognitive processes that are disrupted by frontal lesions or are associated with increased Blood Oxygenation Level-Dependent (BOLD) response in functional magnetic resonance imaging (fMRI) studies have been ascribed a wide variety of names and models such that, “a common functional denominator would appear elusive” (Goldman-Rakic, 1993, p. 13 from Rabbitt et al., 2001). For example, Salthouse (2005) and Davis et al. (2011) both highlight the lack of consensus regarding definitions of ‘executive function’ and the diversity of methods used to assess it. Rabbitt (1997) observed that the common usage of ‘inhibition’ perpetuates misleading analogies between potentially unrelated functional processes.

### 1.2 Uniqueness of theoretical construct

Correlations between test scores for the same theoretical construct “should not be explainable in terms of individual differences in functional property other than the one they are supposed to measure” (Rabbitt et al., 2001, p. 11). Potential confounders of frontal tests may be that they all measure one single construct (e.g. *g*_f_; Duncan, Burgess, & Emslie, 1995) and set of neural sub-systems, or that each test taps multiple latent constructs (also known as *task-impurity*; Miyake and Friedman, 2012, Rabbitt, 1997, Salthouse, 2005) and distinct neural sub-systems. Moreover, strong lesion-symptom double dissociations in the literature remain the exception rather than the rule, and the dense reciprocal connectivity amongst frontal areas has clearly made it difficult to elucidate the specific functional contributions that sub-regions might make. Whereas it is plausible that frontal regions make unique processing contributions to task performance (e.g. Zald, 2007), the current literature might suggest that, at worst, an anatomically pure test of frontal sub-regional function is unattainable (Nyhus & Barceló, 2009), and at best, such a task has not yet been developed (e.g. Manes et al., 2002).

When addressing both criticisms, we propose that taking a neurobiological perspective considerably alters our expectations and interpretation of cognitive test covariances. For example, the following strongly relate to measures of intelligence: putative tests of shifting and working memory (Lehto, Juuarvi, Kooistra, & Pulkkinen, 2003), subtests from the Delis–Kaplan Executive Function System (D-KEFS; Floyd, Bergeron, Hamilton, & Parra, 2010), CANTAB factors of planning and set-shifting (Robbins et al., 1998), Stroop and Tower tests (Crawford et al., 2000, Salthouse, 2005), a factor of updating (Friedman et al., 2006), and a unitary executive function comprised of inhibition, working memory and shifting tests (Brydges, Reid, Fox, & Anderson, 2012). Salthouse also reported that the age effects that were present for the Stroop and Tower tests (Salthouse, 2005) and a variant of the Trail Making test (Salthouse, 2011) were entirely explained by the relationship between age and either reasoning or perceptual speed. The distinct nomenclature (e.g. ‘intelligence’, ‘shifting’, ‘working memory’) sets up an expectation of several unique theoretical constructs, in opposition to the obvious interpretation of these data (i.e. each appear to broadly measure the same construct). Yet, by considering these prior data in light of the proposed neural correlates of *g*_f_ and frontal tests, reported correlations between some neuropsychological tests and general fluid intelligence scores are a realistic expectation because both are consistently linked with common frontal sub-regions (whereas, for other frontal tests and *g*_f_, the converse is true). Dorsolateral prefrontal cortex (DLPFC) and dorsal anterior cingulate cortex (dACC) functioning are implicated in *g*_f_ (Duncan, 2010, Jung and Haier, 2007) and performance on the Tower test (see Methods), Trail Making test (e.g. Yochim et al., 2007, Zakzanis et al., 2005) and stimulus–response conflict tasks such as the Stroop (e.g. Peterson et al., 2002) and Simon tasks (see Methods). By contrast, tests such as the Faux Pas test thought to tap other (non-*g*_f_-implicated) ventromedial frontal regions such as the orbitofrontal cortex (OFC) may not be expected to show such strong associations with intelligence amongst normal, young, healthy populations.

Nevertheless, inferring functional links based on inter-test correlations may be impossible to disconfirm by behavioural evidence alone (Rabbitt, 2011, p. 787), and the concept of the neural substrates of intelligence being confined to the dorsal areas of the frontal lobes has been challenged by several recent findings, discussed below. Thus, extending test correlations to explore whether common variances of ‘frontal’ tasks and intelligence actually have a neurobiological basis in the population under investigation has the potential to more accurately corroborate or disconfirm inferences arising from behavioural evidence. Two studies have taken this approach, both focusing on the correspondence of the loci of brain lesions to scores on frontal tests and intelligence (Barbey et al., 2012, Roca et al., 2010). Both employed a series of tests including components of the D-KEFS (trail-making, verbal fluency, card sorting, twenty questions; Barbey et al., 2012) and the Wisconsin card sorting test, Iowa Gambling Task, verbal fluency and Go-No go (Roca et al., 2010). However, Roca and colleagues arguably included a more diverse range of frontal tasks including the Faux Pas, Hotel and Proverbs tests. Both papers reported that deficits on the majority of these tasks can be mainly explained by a loss of general intelligence. Barbey and colleagues further demonstrated that lesion-symptom mapping for executive and intelligence deficits showed overlapping correspondence to left-sided frontal and parietal lesions. However, Faux Pas, Hotel, and Proverbs scores were not entirely explained by *g* (Roca et al., 2010), and lower scores were related to right frontal lesions. Thus, the limited lesion data might suggest that the frontal neural correlates of intelligence and some common frontal tests pertain to the left frontal cortex, whereas some other components of complex cognition may be less related to intelligence and relate to the right. Yet, to the authors' knowledge, no attempt to relate the correlational structure of intelligence and frontal tests to brain structure has yet been undertaken in generally healthy community-dwelling older people. Such an examination is potentially informative because the pattern of age-related neurostructural change which might explain variance amongst cognitive tests is not uniform across the frontal lobes. Rather, the DLPFC and ACC have been identified as particularly susceptible to age-related atrophy; changes which are hypothesised to drive some age-related changes in cognitive ability (Hedden and Gabrieli, 2004, MacPherson et al., 2002, Tisserand et al., 2002). Thus, the addition of cerebral data can serve to both partially validate behavioural findings and also elucidate frontal loci where significant individual differences in atrophy are behaviourally meaningful.

### 1.3 Summary & study aims

The frontal lobes of the brain are centrally implicated in both general cognitive ability and discrete cognitive processes. It is unclear how frontal tests covary with general intelligence in ageing populations, but it is important to address this question using an appropriate breadth of cognitive tests. Knowledge of the biological bases of test performance in ageing populations is essential because these bases may be different when compared with younger populations with specific lesions, or indeed in healthy younger populations in general, as studied with functional imaging. Some neuropsychological tests have been criticised for i) exhibiting blurred conceptual boundaries and ii) lacking a unique theoretical construct. As discussed above, explanations at the level of cognitive processes may be too ambiguous to interpret the correlational structure of multiple tests of complex cognition using behavioural measures alone. Rather, our interpretation of cognitive test relationships is enhanced if we consider the age-related variance amongst neural substrates of these tests.

In a group of healthy, community-dwelling older adults, this study therefore aims to examine whether – and to what degree – behavioural performance on a selection of tests (suggested as indices of frontal function) overlaps with a measure of *g*_f_ in older age. We also aim to establish how factors of common test score variance relate to individual differences in regional frontal integrity.

Firstly, we examine frontal tests' internal consistency. Tests thought to be sensitive to the functions of the dorsal or ventral frontal lobes should correlate more strongly with each other than with other frontal tests (a test of their *conceptual boundaries*). Secondly, in order to address the *uniqueness of theoretical constructs*, we first aim to consider frontal tests' correlations with a measure of *g*_f_, and then examine whether frontal lobe tests share any unique variance beyond that accounted for by *g*_f_. Consistent with current models of *g*, we would expect *g*_f_ to correlate more strongly with neuropsychological tests sensitive to the dorsal than ventral frontal lobe. Finally, commonality amongst the cognitive measures is explored using principal component analyses. Such a latent variable approach has been suggested as appropriate for partially alleviating the task-impurity problem (Miyake & Friedman, 2012). The extracted principal components are then correlated with frontal lobe sub-regional volumes in order to examine their possible biological foundations in older age. By statistically controlling the volume of frontal lobe sub-regions for intra-cranial volume, the resultant measures indicate the degree to which the raw volume is smaller than would be expected when the brain was at its maximal size (filling the intra-cranial vault). Thus, correlations between these cerebral measures and cognitive components allow us to broadly estimate the frontal loci in which age-related brain changes have occurred that are functionally relevant to a particular aspect of cognition.

## 2 Methods

### 2.1 Participants

The participants were 90 elderly community-dwelling males from the Lothian Birth Cohort 1936 (LBC1936). The members of this cohort were born in 1936 and sat a valid IQ-type test at school in Scotland in 1947 at an average age of 11 years. At around 70 years of age, 1091 surviving, healthy, community-dwelling residents in the Edinburgh area who had taken this initial test were recruited as the LBC1936. The initial wave of testing contained this same mental test in addition to other cognitive and medical tests which are detailed elsewhere (Deary et al., 2007). Three years later, 866 returned for a second follow-up wave of cognitive testing and an MRI scan.

From this second wave, the participants were selected on the following criteria: score of 24 or greater on the Mini-Mental State Exam (MMSE; Folstein, Folstein, & McHugh, 1975), score less than 11 on the depression facet of the Hospital Anxiety and Depression Scale (Zigmond & Snaith, 1983), had an MRI scan less than 1.5 years before cognitive testing, and not taking any antidepressant or glucocorticoid medication. Of the 118 potential participants, 90 agreed to participate. Written informed consent was obtained from each participant and the study was conducted in compliance with departmental guidelines on participant testing and the Declaration of Helsinki. Ethical approval was gained from NHS Lothian Research Ethics Committee (NREC:07/MRE10/58) and the Philosophy, Psychology and Language Sciences Research Ethics Committee at the University of Edinburgh.

### 2.2 Cognitive tests

#### 2.2.1 General cognitive ability factor (*g*_f_)

A general cognitive ability factor (*g*_f_) was derived from the first unrotated principal component analysis (PCA) of the Wechsler Memory Scale — III (WMS-III; Wechsler, 1998 ) UK subtest (Backward Digit Span) and Wechsler Adult Intelligence Scale — III (WAIS-III; Wechsler, 1998) UK subtests (Letter–Number Sequencing, Matrix Reasoning, Block Design, Digit Symbol and Symbol Search). A fuller description of these tests is available in an open-access LBC1936 protocol (Deary et al., 2007).

#### 2.2.2 Tower test (D-KEFS; Delis, Kaplan, & Kramer, 2001)

Successful completion of the Tower test (taken from the D-KEFS) required solving 9 problems, each beginning with wooden discs in a specific configuration on a 3-peg board. Patient studies and functional neuroimaging have implicated the dorsal frontal lobe (dorsolateral prefrontal cortex: DLPFC; anterior cingulate cortex: ACC) in the performance on the Tower test (Boghi et al., 2006, Cazalis et al., 2003, Cazalis et al., 2006, Gomez-Beldarrain et al., 2004, Yochim et al., 2009). The objective of the task is to move the discs such that the participant creates a wooden tower depicted in a target image in as few moves as possible within a specified time limit (which increases as problems become more complex, up to 240 s). The participant may only move one disc at a time, can never place a larger disc on top of a smaller one, and these instructions are displayed throughout, at the foot of the target stimuli. The main outcome variable was Total Achievement Score (/30) in accordance with D-KEFS scoring booklet.

#### 2.2.3 Self-Ordered Pointing Task (SOPT; Petrides & Milner, 1982)

The successful SOPT performance has been related to the integrity of the dorsal frontal lobe (Berlin et al., 2004, de Zubicaray et al., 1997, Petrides et al., 1993, Petrides and Milner, 1982). In the current study, the task was administered using a computerised version, containing a grid of 12 abstract designs (MacPherson et al., 2002) on a touchscreen interface (iiyama ProLite T2250MTS 22 in. 1920 x 1080). The participant was required to select each design only once, choosing an item that they have not previously selected. Testing continued until 12 selections have been made. Following each choice, the order of some of the items in the grid was rearranged to ensure that the participants remember the previously-chosen images by their appearance rather than their location. The test ended after three trials have been completed; these trials involved the same images but in different spatial arrangements, and the trials are self-paced. The outcome measure was the number of times a previously-chosen item was selected and was averaged across the three trials.

#### 2.2.4 Reversal Learning (Rolls, Hornak, Wade, & McGrath, 1994)

Patient and lesion data indicate a central role for the ventral frontal lobe in the performance of this task (Berlin et al., 2004, Cools et al., 2002, Fellows and Farah, 2005a, Ghahremani et al., 2010; Gläscher, Hampton & O'Doherty, 2009; Kringelbach and Rolls, 2003, O'Doherty et al., 2001, Remijnse et al., 2005, Rolls et al., 1994, Wheeler and Fellows, 2008). We used a modified version of a previously-reported neuroimaging paradigm (Hampton & O'Doherty, 2007). We used a deterministic contingency (given the large amount of training required for probabilistic versions) and altered the images from US cents to British pence. The participants were presented with 2 fractal images with the aim of determining which selection will allow them to make the most money. One image will always give a win of 25p, and the other always a loss of 25p. Once the correct image is correctly identified (indicated by 8 consecutive correct selections), the stimulus–reward contingency was reversed. This pattern continued for 50 trials, allowing a maximum of 5 reversals after the initial contingency has been learned. The main outcome variable was the total number of incorrect selections.

#### 2.2.5 Faux Pas test (Gregory et al., 2002, Stone et al., 1998)

This task requires the participants to identify whether a protagonist said something awkward, or something they should not have said in 20 short stories (10 containing a faux pas). Ventral frontal lesions have been reported to impair Faux Pas performance (Lee et al., 2010, Stone et al., 1998; Shamay-Tsoory, Tomer, Berger, Goldsher, & Aharon-Peretz, 2005; Shamay-Tsoory, Tomer, Berger, & Aharon-Peretz, 2003). The participants read the stories at their own pace and were instructed to tell the experimenter when they had finished each one. They were then asked a series of questions about each story to determine whether the participant understood that a faux pas had occurred, including 2 factual control questions to ensure general understanding of the story. All participants exhibited a good factual understanding (M = 39.31, SD = 1.3 out of a possible 40). The story remained in front of the participants at all times. The audio-taped responses were marked in accordance with scoring guidelines (http://www2.psy.uq.edu.au/~stone/Faux_Pas_Recog_Test.pdf). The main outcome measure was the total number of correct responses to questions about the Faux Pas stories (out of a possible 50, excluding the empathy question which asked the participants to describe how the protagonist might feel).

#### 2.2.6 Simon Task (Simon, 1969)

We administered a version of the Simon Task reported by di Pellegrino, Ciaramelli, and Làdavas (2007), translated into English. The participants were required to respond as quickly and accurately as possible to the appearance of a red or green square on a computer screen by pressing the red or green key on the keyboard (positioned on the A and L keys respectively of a QWERTY keyboard). A single square appeared on either the left or the right of the screen, making the required response for a red square incongruent if it appears on the right. The participants were made explicitly aware if they had made an error by the appearance of “(!!!!)” on the screen following their response. The dorsal frontal lobe has been particularly implicated in the neural response to incongruency effects and post-error slowing elicited during such stimulus–response conflict tasks (Botvinick et al., 2004, Cohen et al., 1999, Egner, 2007, Peterson et al., 2002, Swick and Turken, 2002, Ullsperger and von Cramon, 2004). The main outcome variables were the Simon Effect (mean RT on incongruent trials/congruent trials), the directional Simon Effect (mean RT on incongruent trials that follow a congruent trial/congruent trials that follow an incongruent trial; di Pellegrino et al., 2007) and post-error slowing (PES; mean RT on trials following an error/trials with no error).

#### 2.2.7 Dilemmas Task (Greene, Sommerville, Nystrom, Darley, & Cohen, 2001)

The participants were presented with a series dilemmas; each one was followed by a suggested action in order to resolve the situation (e.g. “Would you push the stranger on to the tracks in order to save the five workmen?”) to which the participants had to respond by pressing y (yes) or n (no). Scenarios in which the value of the potential outcome *and* the personal/moral cost of condoning the suggested action are both high are theorised to elicit a high level of conflict. This form of conflict processing is thought to involve the ventral frontal lobe, and lesions to this region lead to faster and more utilitarian responses, compared to non-lesioned controls (Ciaramelli et al., 2007, Koenigs and Tranel, 2007, Moretto et al., 2010). The task was presented on computer and comprised 11 high-conflict scenarios used by Koenigs and Tranel (2007) and initially Greene et al. (2001). Non-moral and low-conflict dilemmas were excluded altogether as 1) the literature suggests that only those containing hi-conflict moral content demonstrates sensitivity to frontal lobe damage and 2) many of these scenarios are not dilemmas at all (Kahane & Shackel, 2008). Progress through the task was self-paced. The main outcome variables were mean time taken to arrive at a decision, and the percentage of suggested actions that each participant endorsed.

### 2.3 MRI acquisition

Structural MRI data were obtained from a GE Signa Horizon HDxt 1.5 T clinical scanner (General Electric, Milwaukee, WI, USA) using a self-shielding gradient set with maximum gradient strength of 33 m/Tm, and an 8-channel phased-array head coil. A high-resolution T1W volume sequence was acquired in the coronal plane, along the hippocampal long-axis (Wardlaw et al., 2011).

### 2.4 MRI analysis

Frontal lobe sub-regions were manually segmented based on a systematic review of existing protocols and their correspondence to the neuropsychological, functional, cytoarchitectonic and hodological literature (Cox et al., 2014). The resultant protocol was applied by one of the authors (SRC) and was highly reproducible (intra-rater Intra-Class Correlation Coefficients > .96; Shrout & Fleiss, 1979) based on 20 hemispheres measured at least 2 weeks apart. Frontal lobe regional gyral volumes (which include gyral grey and white matter) were derived for the following 6 regions per hemisphere: orbitofrontal, dorsolateral, medial superior frontal, dorsal and ventral anterior cingulate, and inferior frontal. These will be abbreviated to OF, DL, MS, dAC, vAC and IF respectively. Movement artefacts were present in anterior slices of 2 MRI scans, leaving 88 with frontal lobe measures. For detailed reproducibility, boundaries and procedural notes, see Supplementary material. Intracranial volume, measured by one of the authors (NAR), included all structures and CSF inside the dura. The lower limit was the axial slice immediately inferior to the inferior limit of the cerebellar tonsils on or above the superior tip of the odontoid process (Valdés Hernández et al., 2012, Wardlaw et al., 2011).

### 2.5 Statistical analysis

Methods used to determine frontal test internal consistency are found in the Supplementary material. Pearson's product–moment correlation tests were performed to examine the relationships between test scores for variables that approximated to a normal distribution. Spearman's rank order correlation tests were used for those that were non-normally distributed. Next, the relationships between frontal tests and general fluid cognitive ability were examined. Tests for significant differences between correlations from the same sample (Williams, 1959) using the cocor package in R (http://cran.r-project.org/web/packages/cocor/cocor.pdf) were used to test predictions that dorsal frontal tasks (i.e. Simon task, Tower task and SOPT) would correlate with *g*_f_ more strongly than ventral frontal tasks (Dilemmas task, Faux Pas task and Reversal Learning). In light of the strong relationship between *g*_f_ and processing speed (*r* (88) = .78, *p* < .001), we also sought to examine the differences in their correlations with frontal tasks. These analyses and the method of deriving a factor score for processing speed can be found in the Supplementary material. Though our alpha level of significance was *p* < .05, we acknowledged the potential for type I error amongst multiple simultaneous comparisons of inter-correlated variables by also considering interpretations at *p* < .01.

Further analyses tested correlations between frontal test scores after partialling out *g*_f_ to identify unique shared variance between frontal test scores that is not in common with *g*_f_. The correlational structure of cognitive performance was further examined using two principal component analyses (PCAs) of frontal test scores, first without and then with the inclusion of *g*_f_, followed by varimax rotation in SPSS 19. Finally, scores were derived for each participant from the rotated principal components using the PCA that included *g*_f_. These were correlated with frontal lobe regional volumes. To test whether these effects were significantly lateralised, we compared the correlation magnitudes (Williams, 1959, as above) of principal components between homotopic frontal regions.

## 3 Results

Summary statistics for the cognitive scores, including measures of internal consistency for the frontal tests appear in Table 1. In general, the variables exhibited acceptable internal consistency (ICCs > .75) including the SOPT which was the test with the lowest value (Cronbach's alpha = .67). Though no participants obtained a perfect score on any task, there was a markedly skewed distribution for the Faux Pas and Reversal Learning tests, indicative of a ceiling effect. The Simon Effect and directional Simon Effect means indicated that response times were longer for incongruent trials, and for contingency switches from congruent–incongruent than incongruent–congruent, as expected.

**Table 1:** Descriptive statistics of test scores and frontal lobe volumes.

	*n*	Mean	SD	Min	Max	Cons.	# trials
Tower	90	17.60	3.95	9	29	78^a^	9
SOPT	88	2.56	0.94	0.67	4.67	.67	3
Faux Pas^b^	90	39.40	7.35	16.09	49	.86^c^	10
RL Errors^b^	87	12.90	7.17	5	28	.89^c^	51
Post-error slowing	88	1.28	0.17	0.91	1.78	.86^c^	d
Simon Effect	89	1.08	0.06	0.94	1.24	.75^c^	120
SE Direction	89	1.05	0.07	0.89	1.20	.81^c^	e
Dilemmas MRT (s)^b^	86	8.70	4.45	2.75	31.31	.82^c^	11
Dilemmas % Endorsement	86	58.14	23.05	0	100	.91^c^	11
*g*	90	0.03	1.14	− 2.47	3.17	–	–
Left DL	88	25,063.73	5676.50	12,205	43,061	–	–
Right DL	88	24,477.3	5441.09	13,254	36,051	–	–
Left dAC	88	3117.16	1259.85	1139	6302	–	–
Right dAC	88	2684.72	968.62	1059	5598	–	–
Left vAC	88	5026.47	1889.26	1452	9197	–	–
Right vAC	88	3990.77	1540.82	1922	9138	–	–
Left IF	88	16,238.06	2962.52	9421	23,846	–	–
Right IF	88	15,715	3128.29	8675	22,070	–	–
Left OF	88	17,580.23	3656.62	10,067	27,624	–	–
Right OF	88	16,598.45	3543.02	9797	26,924	–	–
Left MS	88	7492.52	1786.05	4299	12,197	–	–
Right MS	88	7545.03	1680.60	3932	12,426	–	–

*Note*. All volumes are given in mm^3^, Cons.: internal consistency using ICCs except for SOPT for which Cronbach's alpha is reported — see Supplementary material for details of analysis, SOPT: mean number of repetitions made on the Self-Ordered Pointing Task, RL Errors: total errors on the Reversal Learning Task, MRT: mean reaction time, DL: dorsolateral frontal, dAC: dorsal anterior cingulate, vAC: ventral anterior cingulate, IF: inferior frontal, OF: orbitofrontal, MS: medial superior frontal gyrus.

a Based on a separate sample of 125 participants, 70–79 years, reported in the D-KEFS technical manual.

b Non-parametric variable (Wilcoxon method used).

c Spearman–Brown corrected.

d Mean number of errors = 6.87.

e Of the total 250 trials, the participants encountered a mean of 116 variable-relevant trials, SD = 16.07.

### 3.1 Frontal test correlations

Correlations between cognitive test scores are presented in the lower diagonal of Table 2. Although these indicate a degree of shared variance between some scores for tests typically expected to tap the same subset of regions, there are also similar amounts of variance shared between tests thought to tap different frontal areas. In general, these correlations are small and several would not survive a more stringent alpha threshold. Thus, the test scores amongst our participants did not show the expected correlational patterns that might be predicted from the neuropsychological literature.

**Table 2:** Cognitive test score correlations.

	Tower score	SOPT repetitions	Faux Pas^a^	RL Errors^a^	Post-error slowing	Simon Effect	SE Direction	Dilemmas mean RT^b^	Dilemmas % Endorsement
Tower score	–	− .08	.13	− .27⁎⁎	.00	− .20†	.11	− .07	− .05
SOPT reps.	− .33⁎⁎	–	− .19	.06	.07	− .15	− .07	.07	.00
Faux Pas^a^	.31⁎⁎	− .36⁎⁎	–	− .13	− .02	.11	.08	− .18	− .15
RL Errors^a^	− .37⁎⁎⁎	.20†	− .24⁎	–	− .16	.20†	− .15	.01	.07
PE Slow	.10	− .05	.04	.20†	–	− .15	− .04	.05	.08
Simon Effect	− .22⁎	− .07	.05	.23⁎	− .26⁎	–	− .00	− .23⁎	.14
SE Direction	.10	− .07	.07	− .14	− .04	− .01	–	− .07	− .13
Dilemmas Mean RT^b^	− .16	.16	− .27⁎	.08	− .02	.21†	− .06	–	− .04
Dilemmas % Endorsement	− .05	.01	− .15	.08	.07	.14	− .13	− .03	–
*g*	.51⁎⁎⁎	− .53⁎⁎⁎	.43⁎⁎⁎	− .32⁎⁎	.21†	− .11	.01	− .24⁎	− .02

*Note*. Test score correlations before (lower diagonal) and after (upper diagonal) partialling out *g*_f_. SOPT = Self-Ordered Pointing Task; RL = Reversal Learning; SE = Simon Effect.

a Non-normally distributed (Spearman method used).

b Log-transformed.

⁎ *p* < .05.

⁎⁎ *p* < .01.

⁎⁎⁎ *p* < .001.

† Trend (.08 < *p* > .05).

### 3.2 Frontal test correlations with *g*_f_

Influential neurobiological models of *g* posit that dorsal but not ventral frontal regions comprise part of the ‘*g* network’ (Duncan, 2010, Jung and Haier, 2007). Therefore it was hypothesised that the Simon task, Tower task and SOPT would correlate with *g*_f_ more strongly than ventral frontal tasks (Dilemmas, Faux Pas and Reversal Learning). The correlations are presented in the bottom row of Table 2. The Simon Effect and Dilemmas endorsements did not correlate significantly with *g*_f_, and there was a trend-level association between higher *g*_f_ and greater PES. All other tasks significantly correlated with *g*, and with medium to large effect sizes (.32 to .53, *p* < .05; Cohen, 1988), and with a small effect size with faster mean RT during the Dilemmas task which would not survive at *p* < .01. The correlations between *g*_f_ and frontal tests were mostly higher than the inter-frontal lobe test correlations reported above. This pattern of correlations was not significantly different when using *g* derived without measures of processing speed (Digit Symbol and Symbol Search from the WAIS-III), or when frontal tests were correlated with a measure of processing speed (Table S2).

### 3.3 Partial correlations between frontal test measures, controlling for *g*_f_

In order to explore the degree to which shared variance between the frontal lobe tests reflects variance shared with *g*_f_, correlations between frontal tests were conducted with *g*_f_ partialled out (Table 2, upper diagonal). The majority of previously significant correlations amongst frontal tests were attenuated to non-significance, but there were some instances where frontal lobe tests shared a significant proportion of variance beyond their mutual relationship with *g*_f_. The relationship between Tower score and total Reversal Learning errors remained significant (*rho* (85) = − .27, *p* = .002), as did the correlation between the Simon Effect and Dilemmas mean RT (*r* (83) = − .23, *p* = .031). The Simon Effect also demonstrated *g*_f_-independent correlations at a trend level with the Tower score (*r* (87) = − .19, *p* = .062) and Reversal Learning Errors (*rho* (84) = .20, *p* = .056). However, all but the relationship between Tower and Reversal Learning would not be significant at *p* < .01 and the remaining partial correlations between all other frontal tests did not reach significance.

### 3.4 Principal component analysis

An initial PCA, using frontal test scores alone (Table 3), did not clearly reflect the hypothesised relationship between the frontal lobe tests and their putative sub-regions. Rather it echoed the correlational structure reported above, with loadings from Tower task, SOPT, Reversal Learning, Faux Pas task and Dilemmas endorsements on component 1 (loading values > .3). The first unrotated component accounted for 24% of the data's variance. The scree plot indicated the extraction of 3 factors which were extracted using varimax rotation (to force orthogonal components) and cumulatively explained 53% of the variance (also shown in Table 3). The second component accounted for a further 16%, comprising the Simon Effect and PES and the shared *g*_f_-independent variances amongst frontal tests previously identified in Table 2 (upper diagonal). Component 3 accounted for a further 13% of the variance and had high loadings for the Dilemmas percentage of endorsements (.75) and the directional Simon Effect (− .66); variables which do not correlate with each other or with any other cognitive score. There was also a comparatively lower loading of PES (.36).

**Table 3:** Principal component analysis loadings without and with *g*_f_.

Cognitive test	1st unrotated	Rotated components			1st unrotated	Rotated components		
		PC1	PC2	PC3		PC1	PC2	PC3
*g*	–	–	–	–	**.81**	**.81**	.09	.07
Tower score	**− .69**	**− .64**	.25	− .15	**.69**	**.66**	.23	− .14
SOPT	**.69**	**.73**	.15	.01	**− .70**	**− .73**	.18	.01
Faux Pas	**− .71**	**− .74**	− .06	.00	**.70**	**.72**	− .09	− .01
RL Errors	**.65**	**.60**	− .29	.10	**− .57**	**− .53**	− .26	.15
PE Slowing	**− .30**	− .25	**.60**	**.36**	**.30**	.25	**.59**	**.34**
Simon Effect	.17	− .01	**− .83**	.15	− .16	− .04	**− .83**	.14
SE Direction	− .16	− .10	− .07	**− .66**	.11	.08	− .08	**− .68**
Dilemmas MRT (ms)	**.37**	**.47**	**.44**	− .08	**− .37**	**− .43**	**.46**	− .06
Dilemmas % Endorsed	.15	.03	− .13	**.75**	− .10	− .04	− .12	**.74**
Eigenvalue	2.20	2.12	1.45	1.18	2.71	2.68	1.43	1.19
Explained variance (%)	24	24	16	13	27	27	14	12
Cumulative proportion	–	24	40	53	–	27	41	53

*Note*. Loadings > .3 are shown in bold. SOPT: Self-Ordered Pointing Task, RL: Reversal Learning, PE: post-error, SE: Simon Effect, MRT: mean reaction time.

Introducing *g*_f_ in a second PCA provides information about the correlational structure of the frontal tests and their relation to *g*. The distribution of loadings on the first unrotated component was broadly unchanged, and had a high loading from *g* (.81; Table 3), accounting for 27% of the total test variance. The scree plot suggested the extraction of 3 components whose loadings are shown in Table 3. That pattern and magnitude of the loadings across the three extracted components were not significantly altered when compared to the previous PCA; *g*_f_'s loading was strongest on the first rotated component (.81) and only slightly altered the amount of test variance in comparison to the first PCA. Rotated component 2 showed a low *g* loading (.09), accounting for 14% of total test variance. The similarities between the first and second PCAs strongly suggest that test score variance can be viewed as components of *g*_f_-common and *g*_f_-independent test score variance. Dominant loadings on the latter component are by tasks commonly thought to tap cognitive conflict or inhibitory processes. Though it is usual to ‘name’ principal components based on what they might reflect (and for ease of reference in subsequent analysis), we are mindful of the irony in speculatively applying cognitive nomenclature which we have argued is potentially misleading. Consequently, we shall refer to the components as PC1, PC2 and PC3 for the moment. A factor of processing speed (see Supplementary material) was significantly correlated with PC1 (*r* (77) = .672, *p* < .001), but not with PC2 (*r* (77) = .115, *p* = .311) or PC3 (*r* (77) = .116, *p* = .309). This suggests that PC2 and PC3 represent units of variance that are largely independent of both *g* and processing speed.

### 3.5 Correlations between principal components and frontal lobe regions

**Fig. 1:**
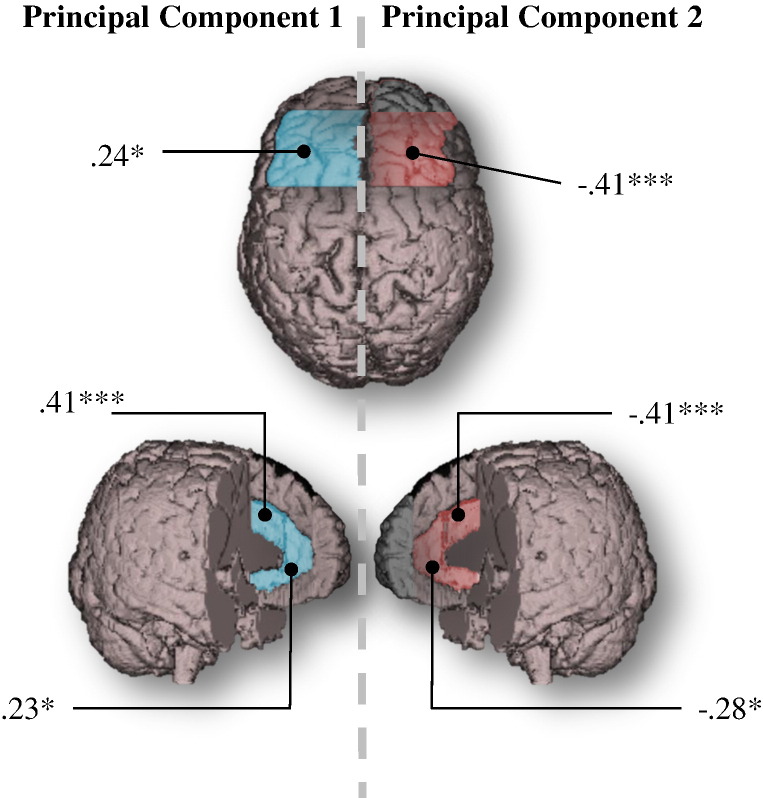
Correlations between frontal lobe regional volumes and principal components 1 (left; blue) and 2 (right; red) derived from frontal test scores and *g*_f_. *p < .05, ***p < .001.

Scores for each of the three rotated components from the second PCA (which includes frontal lobe tests and *g*_f_) were extracted and correlated with brain frontal sub-regional volumes corrected for ICV (Table 4 & Fig. 1). The significant neural correlates of PC1 were left DL, left dAC and left vAC. PC2 significantly correlated with right DL, right dAC and right vAC. PC3 did not significantly correlate with any frontal regional volumes. However, bilateral vAC correlations would not reach significance with a more stringent alpha threshold, nor would that between left DL and PC1.

**Table 4:** Correlations between frontal lobe regional volumes and principal components derived from frontal tests and *g*_f_.

	DL		dAC		vAC		IF		OF		MS	
	L	R	L	R	L	R	L	R	L	R	L	R
PC1	.24⁎	.09	.41⁎⁎⁎	.07	.23⁎	− .03	.20	.19	.10	.00	− .12	− .11
PC2	− .21	− .41⁎⁎⁎	− .05	− .41⁎⁎⁎	.04	− .28⁎	− .04	− .18	.05	− .15	− .20	− .02
PC3	.02	.02	− .10	− .05	− .20	− .12	− .08	− .09	.16	− .05	.09	− .03

*Note*. PC = principal component; DL = dorsolateral; dAC = dorsal anterior cingulate; vAC = ventral anterior cingulate; IF = inferior frontal gyrus; OF = orbitofrontal gyri; MS = medial superior frontal gyrus; L = left; R = right.

⁎ *p* < .05.

⁎⁎⁎ *p* < .001.

Comparing the magnitudes of the correlations between the left and right homotopic brain regions allowed us to test whether the neural correlates of PC1 were significantly left-lateralised, and those of PC2 were right-lateralised. PC1 correlations with dorsal and vAC were significantly greater on the left than the right (*t*(74) = 3.05, *p* = .003; *t*(74) = 2.03, *p* = .046) but the difference between magnitudes for the left and right DL was non-significant (*t*(74) = 1.49, *p* = .141). Correlations between PC2 and brain structure were significantly right-lateralised for the DL (*t*(74) = 2.11, *p* = .039), dAC (*t*(74) = 3.24, *p* = .002) and vAC(*t*(74) = 2.54, *p* = .013). All tests were two-tailed. Correlations amongst principal components and brain regions uncorrected for ICV can be found in the Supplementary material (Table S3). These are similar to the ICV-corrected associations reported herein, albeit with slightly larger effect sizes, likely reflecting the additional contribution of raw head size to cognitive ability.

## 4 Discussion

So-called ‘frontal’ cognitive tests have been criticised for their vagueness of conceptual boundaries, and it is unclear to what extent individual differences in such test scores are explained by so-called ‘general intelligence’ differences in older age. An informative way to proceed, therefore, is to have tests from both neuropsychological and differential psychology traditions, and to have brain information. To this end, we examined the relationships between frontal tests and general intelligence, and interpreted the correlational structure in relation to measures of frontal lobe structure corrected for ICV. The frontal tests did not generally display unique conceptual boundaries, and their relationship with *g*_f_ indicated that they tended to measure two constructs to a greater or lesser degree. Importantly, *g*_f_ accounted for a large degree of variance in frontal tests linked with both dorsal and ventral frontal lobe functioning, in apparent contradiction to influential models of intelligence. The resultant components from the PCA broadly reflected units of *g*_f_-common and *g*_f_-independent variance, and these appeared sensitive to age-related variance in the left and right DL and AC volumes, respectively.

### 4.1 Vagueness of conceptual boundaries

All frontal tests exhibited satisfactory internal consistency, which is important given that correlations between test scores and brain region volumes were the main analyses. The finding that between-region test correlations were of similar or greater magnitude than those within-regions does not support the idea that dorsal and ventral frontal tests exhibit defined conceptual boundaries amongst our group of older participants. Thus – on the strength of behavioural data alone – these frontal tests appear to exhibit blurred conceptual boundaries from the perspective of their putative sub-regional sensitivity amongst our relatively aged participants. This could reflect the issue of task impurity, but might also be because assumptions about tests' sub-regional sensitivity are based on functional imaging data in predominantly younger samples and lesion data, rather than direct evidence in ageing (in which more gradual and widespread atrophic changes occur).

### 4.2 Uniqueness of theoretical construct

In accordance with the previous psychometric literature, *g*_f_ accounted for a large proportion of the variance amongst the frontal test battery. This is illustrated in the pattern of inter-test correlations, the loadings on the first rotated component in our principal component analyses, and the negligible effect that the addition of *g*_f_ had on the factor structure and explained variance (24% to 27%). The split loadings of several tasks across components concur with criticisms of some frontal tests' purity, suggesting that these tests of complex thinking require multiple cognitive contributions from distinct neural substrates.

It was unexpected to find putative tests of dorsal frontal function that showed weak or no significant loading on *g*_f_. Yet there are previous reports of absent correlations between fluid cognition and a factor of inhibition that included a measure of stimulus–response interference (Stroop; Friedman et al., 2006) and PES (Fjell, Westlye, Amlien, & Walhovd, 2012). Whilst the Simon Effect correlates with three other measures from different tasks, the complete absence of any covariance with any other test for both the directional Simon Effect and the Dilemmas percentage endorsement suggests that these variables should be treated with caution. This is corroborated in part by their mutual high loadings on the third principal component.

### 4.3 Neural correlates of principal components

By correlating PC1, PC2 and PC3 with frontal lobe regional volumes, we aimed to understand the portions of cognitive variance in biological terms. This was partially as an additional test of validity, but also because understanding the biological basis of age-related cognitive change is an important step in identifying treatments. It is important to note that this was not an attempt to identify frontal regions that are involved in a given task (which would require different methods). Rather, correcting sub-regional volume for maximal healthy brain size allows us to broadly index the degree of atrophy — that is, we are measuring whether each region is smaller or larger than we would expect, given the brain size prior to age-related atrophy. Correlating these brain measures with cognition therefore allowed us to estimate the functional impact that focal age-related brain changes have had on test scores. The volume of the left AC and DL showed significant correlations with PC1 — this comprises test variance generally accounted for by *g*_f_. Smaller volumes of these regions are therefore characterised by generally poorer test scores (lower *g*_f_, lower Tower score, fewer SOPT errors, a higher Faux Pas score, fewer Reversal Learning errors and faster Dilemmas decision-making).

The right AC and DL exhibited similar correlations with PC2, which contained common variance amongst tests generally independent of *g*_f_. Because the association with PC2 and these brain structures was negative, a smaller right-sided AC and DL reflected generally lower sensitivity to errors (PES) a larger congruency (Simon) effect and faster Dilemmas reaction times. Though the scores with high loadings on PC2 cannot be easily interpreted in terms of how ‘well’ a task is performed, the directions of task loadings on PC2 and the direction of its relationship with frontal brain regions are generally consistent with previous findings. ACC damage has been linked with greater incongruency effects (e.g. Cohen et al., 1999, Ochsner et al., 2001, Swick and Turken, 2002, but see Fellows & Farah, 2005b), and faster Dilemmas responses (Ciaramelli et al., 2007, Koenigs and Tranel, 2007, Moretto et al., 2010). However, a smaller right-sided AC and DL reflected less PES. Though PES is consistently linked with ACC activity, lesion studies do not give a clear indication of how ACC and DLPFC damage might affect PES itself (Modirrousta and Fellows, 2008, Rushworth et al., 2003, Stemmer et al., 2003). Some contend that different types of conflict (e.g. congruency and error) are resolved via partially distinct, multi-stage conflict control loops whose substrates may overlap at the ACC and DLPFC (Egner, 2007, Stemmer et al., 2003, Ullsperger and von Cramon, 2004). Thus, the loading of PES on PC2 and subsequently on some frontal regions may not entirely capture this cognitive process, but the absence of additional subcortical and non-frontal brain information makes us unable to adequately address this.

The absence of any frontal lobe correlations with PC3 confirmed our previous assessment of its composite variables. As predicted, variance in the AC and DL was greater than in other frontal regions. It should be noted that the absence of correlations between any test scores and ventral frontal areas does not contradict theories of their involvement in task processing, but rather indicates that age-related variance is less pronounced and therefore has less cognitive impact.

How might we understand the neural underpinnings of these components in terms of existing theories of cognitive ability? The finding of this partial left–right frontal asymmetry receives support from other empirical evidence of lateralised functional segregation. Two lesion-symptom mapping studies have linked left-sided frontal lesions with intelligence deficits (Barbey et al., 2012; Gläscher et al., 2010) and one has linked test score variance not common to *g* with right sided frontal lesions (Roca et al., 2010). Intelligence in older age was also recently associated with the left but not right dorsal frontal cortical thickness (Karama et al., 2014). Similarly, the right frontolateral activity is related to post-error-related activity (thought to be involved in adaptive post-error behaviour; e.g. Hester, Barre, Mattingley, Foxe, & Garavan, 2007), the resolution of stimulus–response conflict induced by incongruent trials (Milham, 2003) and moral judgement (Tassy et al., 2012). It therefore appears that there is some degree of consistency between our findings and the evidence for hemispheric lateralisation of cognition function.

From cognitive psychology, based on numerous lesion and imaging studies, it has been proposed that the left lateral regions are involved in the capacity to form or select task-relevant rules (criterion setting) and right lateral regions are tasked with updating contingencies and dynamic fine-tuning performance of ongoing behaviours (monitoring) in order to optimise behaviour (Stuss, 2011, Stuss and Alexander, 2007, Vallesi, 2012). Though cognitive nomenclature may result in the blurring of conceptual boundaries, we cautiously observe that the test score loadings on the PCs in our study show a plausible fit. In addition to a high intelligence loading on PC1, performance on the Tower test, SOPT and Reversal Learning all intuitively require the flexible acquisition and selection of rules. It could be argued that the Faux Pas and Dilemmas tasks also require the participant to orient themselves within each scenario and select the appropriate social/moral rules. Likewise, for PC2, performance monitoring and contingency-updating are involved in resolving response conflict from competing stimulus cues (Simon Effect) or conflicting moral perspectives (Dilemmas), and in response to feedback regarding errors where performance may subsequently need optimising (PES).

### 4.4 Limitations and methodological considerations

Psychometrically, the PCA loadings for PC2 are not particularly robust because they show relatively low loadings in general, indicating relatively weak cohesion amongst the common test variances orthogonal to PC1. Nevertheless, the significant correlation of PC2 with frontal lobe structure may indicate that this is a plausible cognitive construct which some of the tasks themselves only measure relatively weakly. In addition, two tasks (Simon Task and Dilemmas task) contributed more than one variable to the analysis. Multiple variables were selected because the literature indicated more than one variable may be related to frontal lobe functioning. However, two of the variables from these tasks (the directional Simon Effect and the percentage of endorsements in the Dilemmas task) had no influence in our analysis and thus their inclusion in the overall results had little bearing. PES and the Simon Effect were both derived from the Simon Task, but they were not strongly correlated, consistent with the view that error detection and conflict processing may be partially dissociable processes, as discussed above. With respect to PC1, it is important to note that the subtests used to derive *g* included two that were explicitly processing speed measures. Though some studies have attempted to empirically separate fluid intelligence and processing speed (e.g. Conway, Cowan, Bunting, Therriault, & Minkoff, 2002), in the Lothian Birth Cohort sample, *g* appears to be fundamentally predicated upon processing speed (Penke et al., 2012). In the current sub-sample, *g*_f_ measures derived with or without overt tests of processing speed (Digit Symbol and Symbol Search) were highly correlated (*r* (88) = .96, *p* < .0001). Removing these measures from our measure of *g* did not affect the observed correlational pattern with frontal tests. Likewise, a measure of processing speed showed the same pattern of results. Moreover, PC1 (heavily loaded on by *g*_f_) was the only component to significantly correlate with processing speed. One may therefore argue that it is unclear whether the relevant overlap with frontal tests is primarily with processing speed or with *g*_f._

Though we aimed to broadly index the degree of age-related atrophic effects in frontal regions (observed size relative to maximal healthy size) on cognition, this is an imperfect measure and sub-regional atrophy is impossible to gauge accurately in a cross-sectional setting. Moreover, declines in brain size and intelligence do not exhibit perfect temporal synchrony; the former showing evidence of decline in the mid-teens (Courchesne et al., 2000) and the latter not until later in the lifecourse (Hedden & Gabrieli, 2004). Thus, part of the observed difference between raw volume and ICV may not reflect age-related atrophic influences.

In addition, our sample size is relatively small compared to studies by Salthouse, 2005, Salthouse, 2011, and is limited to a narrow age range and a relatively healthy male-only sample. Thus, our current design precludes inferences regarding cognitive change over time, its relation to sub-regional frontal lobe structure over time and in a female sample. Whilst these are limitations to the generalisability of our results, they can be considered strengths with respect to the omission of important possible confounders of age and gender. Nevertheless, our self-selecting cohort members are likely to represent a relatively restricted sample; they were representative of the overall LBC1936 cohort whose cognitive abilities were 0.78 of a standard deviation higher than their age group when first tested in 1947, and the variance was restricted by 44% (Johnson et al., 2012). Though Johnson and colleagues found that range restriction did not substantially alter the magnitudes of observed associations, inferences to a broader population should be undertaken with caution. We were also unable to relate the principal components of cognition to other areas of the brain whose structure may be relevant.

Finally, the large number of cognitive tests and frontal brain regions led to a relatively large number of statistical tests. We did not use a conventional correction for multiple comparisons (e.g. Bonferroni), partly due to the exploratory nature of the study and partly because such an approach would be overly conservative. Given the covariances that exist amongst the cognitive tests and also amongst the brain regions under examination, each new comparison does not fulfil the criterion of an independent opportunity for type 1 error to arise (Nyholt, 2001, Ott, 1999). Nevertheless, it is prudent to consider whether our inferences hold if a more stringent significance threshold (e.g. *p* < .01) were applied. Our assessment of a generally weak cohesion amongst the frontal tests and that the majority show some overlap with *g* (Table 2) would remain unchanged, and this would still broadly reflect the loadings observed in PCAs. Regarding the correlations amongst PCs and frontal regions, correlations with the vAC and between PC1 and left DL would be attenuated. Nevertheless, it is not likely for such a pattern of asymmetrical results amongst simultaneous comparisons to arise by chance, and the alpha threshold would not affect our findings of significant lateralisation. Studies with improved power to reliably detect small effect sizes over multiple comparisons are required to corroborate these findings.

In summary, our key findings amongst a group of older adults are as follows: frontal tests exhibited blurred conceptual boundaries, and there was considerable (but not complete) overlap with *g*_f_. A large proportion of test score variance was described by two components which appeared to have discrete neuroanatomical bases in the left and right frontal lobes in older age, indicative of distinct underlying constructs. These constructs may plausibly reflect criterion setting and monitoring referred to in previous reviews of hemispheric lateralisation of function. However, a closer examination of the direction and nature of loadings of individual cognitive tests on the principal components makes a definitive interpretation of this correlational structure challenging, particularly as test scores loading on PC2 cannot easily be gauged by how ‘well’ the task was performed. Whether PC1 represents *g* or ‘criterion setting’ (if such constructs are independent), and PC2 represents ‘monitoring’ is conjecture, but our results indicate that these frontal tasks may require multiple parts of cognition which can be partially explained by their split loadings on these two neuroanatomically distinct frontal components. The addition of cognitive and neurobiological information in older age therefore complements previous lesion work on this subject. Future work could combine structural MRI and cognitive measures to larger ageing samples, with explicit selection of tasks thought to tap these two constructs and it could acquire longitudinal brain imaging data to address the functional implications of change in the same regions amongst individuals over time.

## Appendix A Supplementary data

Supplementary data contains: frontal lobe parcellation boundaries and reliability, details of methods for calculating internal consistency of 'frontal tests', cognitive test score correlations using separately-modeled (orthogonal) measures of g and processing speed, correlations between frontal lobe regional volumes (not corrected for ICV) and principal components.
