# Corrigendum to Correlational structure of ‘frontal’ tests and intelligence tests indicates two components with asymmetrical neurostructural correlates in old age Intelligence 46 (2014) 94–106

**DOI:** 10.1016/j.intell.2018.10.001

**Published:** 2018

**Authors:** Simon R. Cox, Sarah E. MacPherson, Karen J. Ferguson, Jack Nissan, Natalie A. Royle, Alasdair M.J. MacLullich, Joanna M. Wardlaw, Ian J. Deary

**Affiliations:** a Brain Research Imaging Centre, Neuroimaging Sciences, University of Edinburgh, UK; b Centre for Cognitive Ageing and Cognitive Epidemiology, University of Edinburgh, UK; c Department of Psychology, University of Edinburgh, UK; d Geriatric Medicine, University of Edinburgh, UK; e Scottish Imaging Network, a Platform for Scientific Excellence (SINAPSE) Collaboration, UK; f Endocrinology Unit, University of Edinburgh, UK

We reported the incorrect mean, standard deviation and maximum value for one of the cognitive tests reported in Table 1 in the above-mentioned article. The correct values are: Faux Pas test: Mean = 39.29, SD = 7.73, Max = 12.00. We apologise for this oversight.

