# Supplementary material for: Correlational structure of ‘frontal’ tests and intelligence tests indicates two components with asymmetrical neurostructural correlates in old age
Source: Intelligence. 2014 Sep;46:94–106. doi: 10.1016/j.intell.2014.05.006 (PMC4175012; doi:10.1016/j.intell.2014.05.006)
Supplement: Supplementary file 1 — Supplementary data contains: frontal lobe parcellation boundaries and reliability, details of methods for calculating internal consistency of 'frontal tests', cognitive test score correlations using separately-modeled (orthogonal) measures of g and processing speed, correlations between frontal lobe regional volumes (not corrected for ICV) and principal components. [file mmc1.docx]

**Supplementary Material**

**Frontal Lobe Parcellation**

Detailed justification of the protocol described below can be found in:

Cox, S.R., Ferguson, K.J., Royle, N.A., Shenkin, S.D., MacPherson, S.E., MacLullich, A.M.J., Deary, I.J. & Wardlaw, J.M. (2014). A systematic review of brain frontal lobe parcellation techniques in magnetic resonance imaging. *Brain Structure and Function,* 219(1), 1-22.

All parcellation steps were carried out using Analyze 9.0 (Mayo Clinic) and digitizing tablet on T1 weighted images. The first processing step involves applying a rigid-body transform in order to standardise head alignment. This allows cut-planes to be consistently applied across individuals. The transform is applied so that the line between Anterior and Posterior Commissure (AC-PC line) is horizontal in the sagittal plane. The brain is also aligned so that the central fissure is vertical in both coronal and axial views, using the orbits as a secondary guide. In cases where the central fissure is slightly curved in the axial plane, the path of the central fissure in the frontal portion of the brain is used as a preferential guide.

The transformed image is then thresholded using the mean value of sampled intensities of CSF and grey matter from 2 coronal slices posterior to the appearance of the temporal stem (a total of 4 sampling points, 3 x 3 voxels each). The thresholded image is then saved as a separate file and used for the creation of ROI object maps.

The delineation of sub-regions was done by first drawing boundaries into the depth of the relevant sulci. Next, the limit through white matter was established by connecting these sulcal boundaries with a straight line where possible. Where a straight line would pass through grey matter, it was necessary to deviate in order to include this in the ROI. Care was taken to exclude non-brain tissue from measurement, by drawing limits between meningial and brain matter, and to avoid the inclusion of the putamen and other sub-cortical structures. The parcellated regions were then assigned their appropriate ROI using an automated flood-fill, which detected the grey matter - CSF boundary using a threshold (set at the same mean value of sampled intensities calculated during the thresholding procedure above). The ROIs in the following protocol were all traced in the coronal orientation, progressing from posterior to anterior. Viewing and marking of ROIs was also carried out in sagittal and axial planes in order to provide a set of guides during coronal tracing.

The posterior boundary for the superior, middle and inferior frontal gyri can be identified as the coronal slice anterior to the appearance of the precentral gyrus. This has been selected as the boundary primarily to exclude motor and pre-motor regions whilst maintaining an easily-identifiable and reproducible cut-plane. The posterior extent of the orbitofrontal cortex can be determined in the coronal plane as the most anterior slice in which the lateral orbital sulcus appears, allowing differentiation of orbital and insular cortices. At its most dorsal posterior extent, the cingulate gyrus conforms to the dorsal frontal boundary, and runs to its natural limits subgenually, as determined with the help of mid-sagittal views. The frontal pole boundary was the coronal slice immediately anterior to the cingulate gyrus. This was established using a combination of sagittal and coronal views and was used as the anterior boundary for the dorsoalateral, inferior frontal and orbital regions.

The Anterior Cingulate (AC) should be outlined on a sagittal slice near the midline in the first instance. Where the paracingulate gyrus (PCG) is present, this was included in the AC region, thus making the paracingulate sulcus (PCS) the most superior boundary of the ‘AC’ ROI. The tracing of this region should follow the path of the gyrus sub-genually to its clearest sub-callosal and posterior extent.

Moving to the coronal plane at the posterior boundary of the PFC and progressing anteriorly, both dorsal (dAC) and ventral (vAC) regions can be traced, using the markings made from the sagittal slice for guidance. The dAC continues until posterior-most coronal slice in which the hemispheres are still connected by the corpus callosum. From this point forward, all cingulate regions are assigned to the vAC.

The dorsolateral area (DL) comprises the middle frontal gyrus (MFG) and the lateral portion of the superior frontal gyrus (SFG). On the sagittal plane, the SFG can be identified in a slice near the midline, and is limited by the frontal pole and the Anterior Cingulate Sulcus (ACS) or Paracingulate Sulcus (PCS) where present. Returning to the coronal section and starting at the posterior PFC boundary, the SFG can now be traced from posterior-anterior until the appearance of the frontal poles. On the medial wall, the SFG is limited by the ACS or PCS, and is bound laterally by the SFS. This region can then be divided into lateral and medial portions by dropping a line through the crown of the most superior-medial gyrus of this area so that it bisects the roughly horizontal white-matter limit at the base of the gyrus into two equal portions. Often, a straight line may not be possible. In these cases, the line should follow the contours of the white matter branching until a straight line is feasible. The DL’s ventrolateral boundary is the inferior frontal sulcus (IFS) which can be clearly identified in more posterior coronal sections.

The inferior frontal gyrus (IF) comprises all three parts (opercularis, triangularis and orbitalis). The superior boundary is the IFS, and in more posterior coronal slices the IF is bound ventrally by the insula. With more anterior slices however, the insula cortex recedes and the resultant lateral orbital sulcus (LOS) becomes the inferior boundary, differentiating the IFG from orbitofrontal cortex (OF). Tracing continues anteriorly until the frontal poles are reached or the IFS and LOS meet.

The OF can first be identified in the most posterior coronal slice in which insula and OF can be differentiated. Care was taken to exclude subcortical structures and the olfactory bulb. On the medial wall, the OF is consistently bound by the cingulate or paracingulate sulcus. Laterally, the OF is separated from the IF by the LOS, and is bound anteriorly by the frontal pole.

*Table S1*

Reliability of frontal lobe volumetric measurement

| **ROI** | **ICC Agreement** |
| --- | --- |
| IF | 0.96 |
| DL | >0.99 |
| OF | 0.99 |
| MS | 0.99 |
| dAC | 0.98 |
| vAC | 0.99 |
| FP | 0.99 |

*Note.* ROI: region of interest, ICC: intra-class correlation coefficient – performed on repeated measures of 20 hemispheres, at least 2 weeks apart by the same author (SRC), IF: inferior frontal gyrus, DL: dorsolateral, OF: orbitofrontal, MS: medial superior frontal gyrus, d/vAC: dorsal/ventral anterior cingulate, FP: frontal pole.

**Frontal Test Internal Consistency**

The internal consistency of the frontal tests was examined. Within-test items were compared using Cronbach’s Alpha for the SOPT. Intraclass Correlation Coefficients (Shrout, & Fleiss, 1979) were used for the remaining tests, apart from the Tower test which was reported in the Delis-Kaplan Executive Function System (D-KEFS) Technical Manual (Delis, Kaplan & Kramer, 2001), for which normative scores have already been produced using 125 participants, aged 70-79. Coefficients were derived using an odd-even methodology and corrected using the Spearman-Brown formula.

For the Self-Ordered Pointing Task, Cronbach’s Alpha was calculated between the numbers of repetitions made on each of the three trials. For the Faux Pas test, scores for odd and even stories containing a Faux Pas were summed and compared. Split halves of the number of errors during Reversal Learning were created for each participant and compared.

The measure for Post-Error Slowing (PES) in the Simon Task used in the analysis is calculated as the mean reaction time (RT) for trials following an error divided by the mean RT of trials following correct responses. Therefore, consistency was calculated by comparing the mean of odd and even occurrences of PES RTs divided by the mean RT of trials following correct responses for every participant who made more than one error (n=78). In order to ascertain the consistency of the Simon, and directional Simon Effect, a similar procedure to that described for PES was conducted. For the Simon Effect, the mean was of the ratio of RTs on a) incongruent and b) congruent trials. Means for a) were then divided by b). For the directional Simon Effect, this was where the change in contingency went a) from congruent to incongruent and b) from incongruent to congruent. Means for a) were then divided by b) in order to replicate the format used in the analysis, and the internal consistency was calculated by correlating odd and even instances of a/b.

Finally, in the Dilemmas task, both percentage of actions endorsed and the RT data for scenarios were split into odd and even questions of comparable mean emotionality rating (5.70 versus 5.86; ratings reported in Koenigs et al 2007). Spearman-Brown correction (Brown, 1910; Spearman, 1910) was applied to all tests of reliability except the SOPT and Tower.

*Table S2.*

Cognitive Test Score Correlations using separately-modelled factors of g and processing speed.

|  | Speed | Tower Score | SOPT Repetitions | Faux Pas ^a^ | RL Errors ^a^ | Post-Error Slowing | Simon  Effect | SE Direction | Dilemmas Mean RT ^b^ | Dilemmas % Endorsement |
| --- | --- | --- | --- | --- | --- | --- | --- | --- | --- | --- |
| *g* | **.78***** | **.52***** | **-.50***** | **.49***** | **-.33**** | .19 | -.17 | -.02 | **-.23*** | -.03 |
| Speed | **-** | **.45***** | **-.45***** | **.29**** | **-.28**** | **.32**** | -.10 | .10 | **-.24*** | .00 |

*Note. g* is derived using the previously-described PCA method with the exclusion of the two speeded tasks (Digit-Symbol coding and Symbol Search). Speed is derived using the first unrotated solution of a PCA of Digit-Symbol coding, Symbol Search, simple and four-choice reaction time and inspection time (as described in Deary et al., 2007). ^a^ non-normally distributed (Spearman method used)**,** ^b^ log-transformed, *p<.05, **p<.01, ***p<.001, SOPT = self-ordered pointing task; RL = reversal learning; SE = Simon effect.

*Table S3.*

Correlations between frontal lobe regional volumes (not corrected for ICV) and principal components derived from frontal tests and g_f_.

|  | DL | | dAC | | vAC | | IF | | OF | | MS | |
| --- | --- | --- | --- | --- | --- | --- | --- | --- | --- | --- | --- | --- |
|  | L | R | L | R | L | R | L | R | L | R | L | R |
| PC1 | **.24*** | .12 | **.36***** | .13 | **.24*** | .05 | .21 | .20 | .04 | -.09 | -.09 | -.08 |
| PC2 | **-.30**** | **-.47***** | -.16 | **-.48***** | -.08 | **-.33**** | -.14 | **-.26*** | -.10 | **-.24*** | **-.26*** | -.10 |
| PC3 | .06 | .07 | -.04 | -.02 | -.06 | -.17 | -.03 | -.05 | .18 | -.00 | .11 | -.00 |

*Note.* PC = principal component; DL = dorsolateral; dAC = dorsal anterior cingulate; vAC = ventral anterior cingulate; IF = inferior frontal gyrus; OF = orbitofrontal gyri; MS = medial superior frontal gyrus; L = left; R = right. **p*<.05, ***p*<.01, ****p*<.001
